# Two Forms of Social Inequality in Students' Socio-Emotional Skills: Do the Levels of Big Five Personality Traits and Their Associations With Academic Achievement Depend on Parental Socioeconomic Status?

**DOI:** 10.3389/fpsyg.2021.679438

**Published:** 2021-07-21

**Authors:** Clemens M. Lechner, Jens Bender, Naemi D. Brandt, Beatrice Rammstedt

**Affiliations:** ^1^GESIS–Leibniz Institute for the Social Sciences, Mannheim, Germany; ^2^Department of Psychology, University of Hamburg, Hamburg, Germany

**Keywords:** socio-emotional skills, Big Five, personality, social inequality, socioeconomic status, academic achievement, GPA

## Abstract

Some researchers and policymakers advocate a stronger focus on fostering socio-emotional skills in the hope of helping students to succeed academically, especially those who are socially disadvantaged. Others have cautioned that this might increase, rather than reduce, social inequality because personality traits conducive to achievement are themselves unevenly distributed in disfavor of socially disadvantaged students. Our paper contributes to this debate. Analyzing representative, large-scale data on 9,300 ninth graders from the German National Educational Panel Study (NEPS) and using the Big Five personality traits as a measure of socio-emotional skills, we cast light on two related yet distinct aspects of social inequality in socio-emotional skills: First, do *levels* of personality traits conducive to achievement vary as a function of students' parental socioeconomic status (pSES)? Second, do the *returns* to personality traits in terms of trait–achievement relations vary as function of pSES? Results showed that differences in Big Five traits between students with different pSES were small (0.04 ≤ |*r|* ≤ 0.09), especially when compared with pSES-related differences in cognitive skills (fluid intelligence) and sex-related differences in personality. The returns to Conscientiousness—the personality trait most relevant to achievement—in terms of its relations to academic achievement were higher in higher- vs. lower-SES students. Trait–achievement relations did not vary as a function of pSES for the other Big Five traits. Overall, both types of inequality were limited in magnitude. We discuss the implications of these findings for policy and practice and delineate directions for further research.

## Introduction

Fostering socio-emotional skills—which are often conceptualized according to the Big Five framework of personality traits (Abrahams et al., 2019)—through school-based programs and similar interventions has been welcomed as a possible conduit for improving students' academic achievement and life outcomes more generally (e.g., Kautz et al., 2014; Sánchez Puerta et al., 2016; Chernyshenko et al., 2018; Bleidorn et al., 2019; Malanchini et al., 2019). Both researchers and policymakers have espoused the hope that fostering socio-emotional skills particularly among socially disadvantaged students might be a way to reduce social inequality in academic achievement and related outcomes (Damian et al., 2015; Arias et al., 2017; Sisk et al., 2018; Grosz et al., 2021).

Such hopes are founded on the observation that personality traits have robust links to school achievement and other life outcomes (e.g., Roberts et al., 2007; Poropat, 2009; Gutman and Schoon, 2013; OECD, 2015; Borghans et al., 2016; Lechner et al., 2017; Soto, 2019) and may change through educational experiences (Göllner et al., 2017; Brandt et al., 2019). Additionally, these hopes rest on two often implicit assumptions: first, that students from a lower-SES background are at a disadvantage compared with their higher-SES peers when it comes to levels of personality traits such as Conscientiousness and Openness, just as they are when it comes to cognitive skills measured through standardized tests (Damian et al., 2015; Spengler et al., 2015); second, that personality traits conducive to achievement might have greater benefits for students from lower-SES backgrounds (Shanahan et al., 2014; Damian et al., 2015) and might thus compensate for social disadvantage.

The extent to which these assumptions hold is critical in determining whether intervention programs that aim to reduce social inequality by fostering skills and traits conducive to achievement can live up to their promise. However, whereas the links between personality and school achievement are well-established, these additional assumptions have received little research attention. Only few studies have examined the interplay of personality, parental socioeconomic status (pSES), and academic achievement (for an overview, see Ayoub et al., 2018).

In the present study, we contribute to this debate by casting new light on two types of social inequality in students' socio-emotional skills that correspond to the two assumptions mentioned above: (1) *differential levels* of socio-emotional skills related to students' parental socioeconomic status (pSES); and (2) *differential returns* to socio-emotional skills in terms of associations between the Big Five and academic achievement related to students' pSES. We focus on relations between pSES as measured by the highest International Socio-Economic Index (HISEI) of occupational status (Ganzeboom et al., 1992), Big Five personality traits as a global measure of students' socio-emotional skills, and school achievement as measured by grade point average (GPA). For this purpose, we use large-scale data representative of ninth-grade students in Germany.

## Socio-Emotional Skills and School Achievement

Like most previous studies, we use the Big Five model of personality as an organizing framework for conceptualizing socio-emotional skills. The Big Five is the dominant model of individual-difference traits (i.e., typical patterns of thoughts, behaviors, and emotions; John et al., 2008). The Big Five personality traits can be viewed as human capital, skills, or resources because (1) they are conducive to achievement and attainment, and (2) they represent relatively consistent patterns of behavior, cognition, and emotion that are shaped in part by socialization and learning, despite often substantial heritability (Vukasović and Bratko, 2015; Kandler and Zapko-Willmes, 2017).

A sizable body of evidence attests to the relevance of the Big Five personality traits for school achievement. Meta-analyses (Poropat, 2009; McAbee and Oswald, 2013) and large-scale studies (e.g., Borghans et al., 2016; Lechner et al., 2017) show that Conscientiousness and Openness, in particular, are related to better academic achievement—above and beyond cognitive ability. Meta-analytic effect sizes in Poropat (2009) were *r* = 0.24 for Conscientiousness and *r* = 0.09 for Openness after adjusting for cognitive ability. The other three Big Five traits had smaller and more varied associations with achievement—the correlation between Agreeableness and achievement, for example, was *r* = 0.07 [but see Brandt et al. (2020)]. These findings have stimulated interventions to improve student outcomes by fostering traits conducive to school achievement—particularly traits from the Conscientiousness family—thus far with mixed success (Arias et al., 2017; but see Alan and Ertac, 2018; Alan et al., 2019, and Sisk et al., 2018, for encouraging findings).

## Social Inequality in the Levels of Socio-Emotional Skills

In marked contrast to the links between pSES and cognitive ability and achievement, and the links between personality and achievement, the associations between pSES and offspring personality traits have been rarely directly investigated. As Ayoub et al. (2018, Study 1) noted, pSES–personality links were typically not the focus of the studies summarized in their recent meta-analysis. Instead, they were only incidentally reported, for example, because pSES or personality were included as covariates in analyses with a different substantive focus. Consequently, there is a dearth of theoretical groundwork specific to the pSES–personality interface.

Notwithstanding this dearth, there are several more general theoretical arguments why pSES should be related to offspring personality traits (see also Ayoub et al., 2018). The first argument is that pSES shapes the developmental contexts in which children are raised and thereby also their personality. For example, children with lower pSES are, on average, exposed to home environments that are less cognitively stimulating, that are marked by lower parental involvement, greater stress and heightened conflict, and that are situated in less affluent and secure neighborhoods whose less well-funded schools do not offer the same educational opportunities as those attended by higher-SES children (e.g., Bradley and Corwyn, 2002; Kiernan and Huerta, 2008; Donkin et al., 2014; Ryabov, 2020). This multitude of contextual influences may, in turn, shape personality traits in the same way they shape cognitive ability, achievement, and aspirations (e.g., Becker et al., 2012; Damian et al., 2015; Guill et al., 2017). The notion that lower-pSES children are less likely to develop traits conducive to achievement is called the structural amplification hypothesis (Shanahan et al., 2014). Similar ideas are foundational to the family stress model (Conger and Conger, 2002; Masarik and Conger, 2017) and the family investment model (Conger and Donnellan, 2007; Sohr-Preston et al., 2013). Building on these ideas, perhaps the two most plausible consequences of heightened stress and lower cognitive stimulation among lower-SES children are lower levels of Openness to Experience and Conscientiousness and lower levels of Emotional Stability compared with higher-SES children.

Another argument for why pSES and offspring personality might be related draws on behavioral genetics. Personality traits are strongly heritable (Vukasović and Bratko, 2015), and heritability appears to increase with age (Zheng et al., 2019). Children's educational achievement and attainment are also heritable (e.g., Demange et al., 2020), as are specific personality traits conducive to higher educational achievement (e.g., Tucker-Drob et al., 2016; Malanchini et al., 2019; Mõttus et al., 2019). This leads to the notion that children inherit genes from their parents that shaped parents' personality and SES and that, in turn, shape childrens' personality as well as their achievement and later-life SES. Thus, whereas the first argument assumes social causation of personality differences by pSES through contextual effects on development, the second assumes genetic causation as the main reason for the pSES–personality association. Of course, as the developmental systems perspective highlights, social and genetic causation are neither mutually exclusive nor independent, but closely intertwined through gene–environment correlation and interaction as well as through epigenetic processes which, in turn, co-act with individual agency (Ford and Lerner, 1992; Lerner and Overton, 2017; see also Roberts, 2018).

Existing evidence, albeit sparse, provides qualified support for the idea that pSES, and personality traits are related. A recent meta-analysis by Ayoub et al. (2018) found that higher pSES was linked to higher offspring Openness (*****r***** = 0.14). Associations with other personality and temperament traits were small, leading these authors to conclude that Openness is the only personality trait that shows relevant pSES-related inequality. However, effect sizes varied widely across studies. Sutin et al. (2017) tested the association between parents' educational levels and offspring personality traits in 7 samples (age range 14–95 years) and meta-analytically combined the results. They found that parental educational attainment was positively related to offspring Openness, Extraversion, and Emotional Stability. Associations between pSES and offspring personality were the same for adopted and biological children, underscoring environmental/behavioral influences of pSES on personality, and supporting social causation. Sutin et al. (2017) argued that a higher income enables parents to provide more diverse experiences to their offspring, which fosters the development of their Openness. Unexpectedly, as in Ayoub et al. (2018), there was no association between parental education and offspring Conscientiousness, save a *negative* association in younger cohorts.

## Social Inequality in the Returns to Socio-Emotional Skils

Even fewer studies have analyzed possible interactions between pSES and offspring personality traits. Such interactions could reveal whether the returns to traits such as Openness and Conscientiousness with regard to school achievement are the same or different for children from lower- vs. higher-SES families. Most research on pSES, personality, and achievement has focused on linear effects of personality on achievement (for a meta-analysis, see Poropat, 2009). Further studies have investigated personality as a mediator in the pSES–achievement relation. Steinmayr et al. (2010), for example, found that Openness, and to a lesser extent Conscientiousness, mediated the association between parent's education and students' grades in the academically most demanding school track in Germany. Only more recently have studies explored differential returns to personality traits for achievement depending on factors such as grade level (e.g., Vedel and Poropat, 2017), school subject (Spengler et al., 2013; Israel et al., 2019; Meyer et al., 2019; Brandt et al., 2020), school track (Brandt et al., 2020), and pSES (Ayoub et al., 2018, Study 2). Theoretical work specific to interactions between pSES and offspring personality is in commensurately short supply.

Two general theoretical perspectives exist on such interactions in research on SES and life outcomes (see also Damian et al., 2015). The first is the resource substitution hypothesis (Mirowsky and Ross, 2003), which states that individual characteristics (including, perhaps, personality traits) can compensate for structural disadvantages that flow from a lower pSES. This implies *compensatory* interactions whereby achievement may be high for lower-SES students if they possess high levels of personality traits conducive to achievement. Conversely, achievement may be high even in the absence of favorable personality traits if pSES is high. In the former case, higher Conscientiousness (i.e., self-discipline, industriousness), for example, may compensate for a lack of structure and parental involvement in a low-SES household.

The opposite view is the resource amplification or “Matthew effect” hypothesis (Walberg and Tsai, 1983; see also Blossfeld and von Maurice, 2011). It holds that structural characteristics such as a higher SES and individual characteristics such as higher levels of achievement-related personality traits coalesce in producing life outcomes. Applied to the present case, this perspective would predict *synergistic* interactions, whereby achievement is highest when both pSES and personality traits conducive to achievement are high. This implies that children from higher-SES backgrounds, who are already socially privileged, would benefit disproportionately from having the “right” traits.

There is evidence, albeit scarce, to support each of these competing views. Shanahan et al. (2014), for example, found evidence for the resource substitution hypothesis in adolescent middle and high school students in the United States. Students from a lower socioeconomic background were more likely to attain a higher level of educational attainment if they had higher levels of Agreeableness, Openness, and Emotional Stability. At the same time, higher levels of these traits were less likely among students from low-SES households. In another study, Damian et al. (2015) found some evidence for compensatory effects of Extraversion and Conscientiousness in U.S. high school students; these effects were robust when controlling for cognitive ability. The authors found support for the Matthew effect only in terms of cognitive ability, but not personality. Analyzing a very large but selective online sample, Ayoub et al. (2018, Study 2), found synergistic interactions between parental education and all Big five traits, although these interactions were small in size.

In the German context, which is the focus of the present study, Brandt et al. (2020) investigated whether personality–achievement relations differ depending on school tracks in the historically three-tiered German secondary school system. They found that Conscientiousness and Agreeableness, and partly also Openness, were more strongly related to academic achievement in mathematics and German in the highest (academically oriented) track, in which higher-SES students are concentrated, than in the lowest (vocationally oriented) track, in which lower-SES students are prevalent. The interactions they found were more in line with the resource amplification (Matthew effect) hypothesis than with the resource substitution hypothesis. Although that study did not investigate pSES but school tracks as a moderator of personality–achievement relations, the school tracks to which students in Germany are assigned after primary school strongly depend on pSES—a fact that researchers have long deplored (e.g., Baumert et al., 2006; Maaz et al., 2008; Chmielewski et al., 2013; Chmielewski and Reardon, 2016). Thus, it is conceivable that the differential returns to personality traits by school track observed in Brandt et al. (2020) partly reflect differential returns related to pSES. The interplay between between-school tracking and pSES as moderators of the personality–achievement relations is currently poorly understood.

Although the resource substitution and resource amplification perspectives are mutually exclusive for a single trait, they may both be true but for different traits. For example, a study on personality traits as predictors of successful educational transitions from school to work (but not school achievement) in Germany found mainly synergistic interactions between pSES and Openness but also a few compensatory interactions with other traits (Nießen et al., 2020).

In sum, little research has addressed possible interactions between pSES and offspring personality, and extant findings are inconclusive. The verdict is not yet in on which of the two perspectives—resource substitution or resource amplification—more accurately describes how pSES and personality interact in predicting achievement, particularly in the German school system, where students are tracked into educational pathways early in their school lives (generally at age 10).

## Aims and Research Questions of the Present Study

In sum, existing evidence shows that pSES is related to children's personality traits, especially to Openness—and apparently both through social causation and shared genetic influences (Sutin et al., 2017; Ayoub et al., 2018, Study 1). Moreover, there is evidence—albeit very scarce—to suggest that the returns to personality traits differ as a function of pSES. However, existing evidence varies widely with regard to the type and quality of personality and outcome measures, size and composition of samples, study design, and other factors (see overview in Ayoub et al., 2018, Study 1). Hence, it is unclear how these factors affected the pSES–personality estimates in these studies. Moreover, most prior research hails from the North American context and is based on small and non-representative samples (for a notable exception, see Sutin et al., 2017). No previous work has investigated whether mean-levels or returns to personality traits differ by pSES in representative German samples (but see Steinmayr et al., 2010, for a small-scale study in Germany).

Our present study adds to the body of evidence on the interplay between pSES, personality, and achievement. Using large-scale representative data on ninth-grade students from the German National Educational Panel Study (NEPS), we seek to answer two questions that to date have only partly been resolved. First, do students from higher- and lower-SES families differ in personality traits that are conducive to achievement (social inequality in the levels of socio-emotional skills)? While we are cognizant of the genetic entwinement of pSES, personality, and achievement, here we build on the family stress and family investment models (Conger and Donnellan, 2007). We hypothesize that the two personality traits that are most conducive to achievement, Conscientiousness, and Openness (Poropat, 2009), are higher in students from more socially advantaged families. We also expect Negative Emotionality to be lower and Extraversion and Agreeableness to be higher in higher-SES students. As points of comparison, we report sex-related differences in Big Five personality traits (i.e., another well-known predictor of personality differences; Schmitt et al., 2008) and pSES-related differences in cognitive ability (i.e., another student outcome predicted by pSES; Damian et al., 2015; Spengler et al., 2015). Although not the focus of our investigation, including these effects will make it easier to interpret the magnitude of any pSES-related differences we find.

Second, do the returns to personality traits in terms of their links to school achievement (i.e., GPA) vary as a function of pSES? In other words, are personality traits differentially related to achievement in students from higher- vs. lower-SES families? Given the inconclusive prior evidence on this issue, we have no apriori hypotheses. We will test interactions between pSES and personality in an exploratory fashion and examine whether any potential interactions resemble the resource substitution or resource amplification (Matthew effect) patterns.

In addressing this second research question, we also pay heed to the close connections between pSES and the school tracks to which students are assigned in the German educational system. As noted earlier, Germany is a context in which students are clustered into relatively homogenous learning groups after primary school according to their previous achievement (e.g., Chmielewski et al., 2013). Research shows that school tracks moderate personality–achievement relations in Germany (Brandt et al., 2020), and that between-school tracking can increase social inequality because the tracks to which students are assigned after primary school depend strongly on pSES (Baumert et al., 2006; Maaz et al., 2008; Chmielewski and Reardon, 2016). We will therefore control for school track and its interactions with personality.

## Materials and Methods

### Sample

We used data from Starting Cohort 4 of the German National Educational Panel Study (NEPS), an ongoing multi-cohort panel study on educational trajectories and returns to education in Germany (Blossfeld et al., 2011). Starting Cohort 4 comprises a representative sample of 16,425 ninth graders in German secondary schools. The present analyses used data from Waves 1 and 2, which were collected in the form of paper-and-pencil interviews (PAPI) some months after the beginning and near the end of ninth grade (November 2010–January 2011 and May 2011–July 2011, respectively).

We excluded students from special schools and from schools that do not give grades (Waldorf schools), as well as students with missing values or unclear information on their school tracks (*n* = 2,032). We further excluded students with missing values on any (or on a combination) of the other relevant study variables—that is, personality (*n* = 2,074), pSES (*n* = 4,350), academic achievement (*n* = 1,912), cognitive ability (*n* = 2,472), sex (*n* = 880), and migration background (*n* = 1,920). The final sample size for the complete case analysis consisted of *N* = 9,300 students. The average age at Wave 1 was 15.1 years (*SD* = 0.60); 51.8% were female.

### Measures

Supplementary Table 1 provides descriptive statistics and correlations of all study variables.

#### Personality

The Big Five personality traits were assessed with the BFI-10 (Rammstedt and John, 2007), a short version of the Big Five Inventory measuring each Big Five dimension with one positively keyed and one negatively keyed item, plus an additional item for Agreeableness (i.e., 11 items in total). The BFI-10 was administered in Wave 1. Students rated all items on a 5-point scale ranging from 1 (*does not apply at all*) to 5 (*fully applies*). Using positively and negatively keyed items removes bias from acquiescent responding (“yeah-saying”), a response style often observed in individuals with lower SES or lower cognitive ability (e.g., Lechner and Rammstedt, 2015).

Earlier studies supported the retest reliability of the BFI-10 scales as well as its convergent validity with longer scales (Rammstedt and John, 2007; Rammstedt et al., 2020). Reliabilities in the present sample (column “internal consistency” in Supplementary Table 1) were satisfactory. Moreover, through latent measurement models, we established that a model with five factors and an acquiescence factor (as in Brandt et al., 2020) had good fit to the data and showed (partial) scalar measurement invariance across students from different pSES quartiles. For details, see Supplementary Tables 2, 3.

#### Parents' Socioeconomic Status

We used the highest International Socio-Economic Index (HISEI) of occupational status (Ganzeboom et al., 1992; Ganzeboom, 2010), assessed in Wave 1, as a measure of pSES. In line with the international and national assessment standards, students described their parents' occupation (“What do your parents currently do? E.g., car mechanic, sales clerk, high school teacher, civil engineer”) in responses to two open-ended questions, one referring to the mother's and one to the father's occupation. Responses to these questions were coded based on the International Standard Classification of Occupation 2008 (ISCO-08; ILO, 2012). Next, the ISCO-08 code of each parent separately was assigned an ISEI-08 score (Ganzeboom, 2010). ISEI-08 ranks occupations on a scale from 10 (e.g., kitchen helpers) to 90 (e.g., judges) based on the average level of education and average earnings of job holders (Ganzeboom, 2010). The HISEI-08 score was calculated by selecting the higher of the two parents' ISEI-08 scores. HISEI is a well-established measure in educational studies such as the Programme for the International Student Assessment (PISA; OECD, 2019).

#### Academic Achievement (GPA)

We assessed academic achievement via school grades. In Wave 2, students were asked to report their last mid-year report card grades in German, mathematics, physics, chemistry, and biology (or natural sciences, a school subject in some federal states that combines physics, chemistry, and biology). We computed the GPA across these six school subjects (Cronbach's α = 0.87). In Germany, academic achievement ranges from 1 (*very good*) to 6 (*failed*). To facilitate interpretation, we inverted the academic achievement such that higher values corresponded to higher achievement.

#### Additional Variables

The additional variables included in our analyses were cognitive ability, school track, sex, and migration background. We included sex and cognitive ability in order to compare any pSES-related differences we found against sex-related differences in personality as well as against pSES-related differences in cognitive ability. This allowed for a more meaningful interpretation of pSES-related differences in the Big Five. Moreover, we included cognitive ability, sex, school track, and migration background in our analyses regarding the second research question (returns to personality traits) in order to, again, compare effect sizes of personality against these other variables; and to control for potential confounders of the personality–achievement association and the interactions between personality and pSES. Note that controlling for these additional variables provides conservative estimates of the personality–achievement relations because personality traits may be partial mediators of the links between gender, migration background, and school track and achievement.

Cognitive ability was measured in Wave 2 with the NEPS matrices test (NEPS-MAT), an indicator of reasoning ability (fluid intelligence). NEPS-MAT is a 12-item matrices test similar to Raven's Standard Progressive Matrices. Each item comprises a matrix of different geometrical elements with one field remaining free. Respondents have to deduce the logical rules on which the pattern of geometrical elements is based in order to select from the options provided the correct element for the free field. The items are scored as 1 (*solved*) or 0 (*not solved*); we used the sum score across all 12 items, ranging from 0 to 12 (Cronbach's α = 0.66).

Within Germany's historically three-tiered secondary school system (*Hauptschule, Realschule*, and *Gymnasium*), today there are many different school types. In all federal states, the *Gymnasium* (or a *Gymnasium* stream within another school track) gives direct access to tertiary education (university/university of applied sciences), whereas the lower school tracks, *Hauptschule* and *Realschule* (or their respective streams in other school types) do not. Therefore, we grouped the various German school tracks into academically oriented school tracks (*n* = 3,996; *Gymnasium* and *Gymnasium* streams) and vocationally oriented school tracks (*n* = 5,304; *Hauptschule, Realschule*, and their respective streams in other school types). After primary school, students are selected into academically oriented or vocationally oriented school tracks based largely on their prior academic achievement. While students in academically oriented tracks typically have 12–13 years of schooling and often transition to tertiary education, students in vocationally oriented tracks typically have 9–12 years of schooling and often transition to vocational education and training.

In line with the literature (e.g., OECD, 2019), we assessed migration background via students' self-reports of their own and their parents' country of birth at Wave 1. We distinguished between students without a migration background (i.e., student and both parents born in Germany) and students with a migration background (i.e., student and/or at least one parent born abroad).

### Statistical Analyses

We analyzed the two types of social inequality in social-emotional skills (differential levels and differential returns) as follows: First, we examined whether students' mean levels of personality traits (Big Five) differed depending on their pSES (HISEI). We tested this research question in two ways. On the one hand, we examined linear correlations of the Big Five dimensions with pSES (treated as a continuous variable). On the other, hand, we examined mean-level differences by pSES group. To do so, we performed a quartile split on the HISEI variable to obtain four equally sized pSES groups and analyzed the mean-level differences in each of the Big Five dimensions using analyses of variance (ANOVA). The analyses by quartile provided an opportunity to quantify pSES-related differences as *group* differences, which may be more readily interpretable than a linear correlation.

Second, we investigated whether the associations between personality traits and achievement (GPA) differed depending on pSES (HISEI). The parameter of interest here are the interactions between each of the Big Five traits and HISEI. For this purpose, we initially regressed academic achievement on the students' (*z*-standardized) Big Five traits, HISEI (standardized) and the Big Five × HISEI interactions (Model I). In a next step (Model II), we additionally incorporated school track, sex, and migration background as well as their respective interactions with the Big Five and cognitive ability. This allowed us (1) to gauge the extent to which the differential returns to the Big Five were unique or could be explained by fundamental sociodemographic characteristics, and (2) to compare the (differential) returns to personality with those to cognitive ability. To do so, fluid intelligence (standardized), sex (dummy coded: 0 [*female*], 1 [*male*]), migration background (dummy coded: 0 [*no*], 1 [*yes*]), school track (dummy coded: 0 [*academically oriented*], 1 [*vocationally oriented*]), the Big Five × school track, and the intelligence × school track interactions were added to the model.

For collinearity diagnostics, we computed the variance inflation factor (VIF) for our regression models. VIF was consistently below 5. More precisely, the VIF of the Model I predictors ranged between 1.02 and 1.09 and the VIF of the Model II predictors ranged between 1.05 and 4.62. This demonstrates that multicollinearity was not an issue in our analyses.

## Results

### Differential Levels of Personality Traits

Do students' personality traits differ depending on their pSES? As shown in Table 1, we found small correlations between the Big Five and HISEI. Students with higher pSES reported lower (not higher) Conscientiousness, higher Openness, Extraversion, and Emotional Stability, and lower Agreeableness than students with lower pSES. Effect sizes were all small in size (0.04 ≤ |*r*| ≤ 0.09), below the 20th percentile of correlations in individual-differences research (Gignac and Szodorai, 2016). The largest correlations of pSES were those with Openness, *r* = 0.09, 95% CI [0.07, 0.11]; Conscientiousness, *r* = −0.07, 95% CI [−0.05, −0.09]; and Extraversion, *r* = 0.07, 95% CI [0.05, 0.09]; followed by Emotional Stability, *r* = 0.06, 95% CI [0.04, 0.08], and Agreeableness, *r* = −0.04, 95% CI [−0.06, −0.02].

**Table 1:** Mean differences in personality (Big Five) and cognitive ability (fluid intelligence) related to pSES.

	**Total**		**Differences related to parental socioeconomic status (pSES, as measured by HISEI)**						
	**(*****N*** **= 9,300)**		**Very low 1st quartile**	**Rather low 2nd quartile**	**Rather high 3rd quartile**	**Very high 4th quartile**		**4th quartile vs. 1st quartile**	**Linear correlation with pSES**
	**Range**	***M* (*SD*)**	***M* (*SD*)**	***M* (*SD*)**	***M* (*SD*)**	***M* (*SD*)**	**η*^**2**^***	**Cohen's *d***	***r***
**Personality**									
Conscientiousness	1–5	3.17 (0.88)	3.23 (0.86)	3.22 (0.87)	3.18 (0.88)	3.06 (0.88)	0.006	−0.20	−0.07
Openness	1–5	3.50 (0.94)	3.40 (0.90)	3.46 (0.94)	3.53 (0.97)	3.59 (0.95)	0.006	0.20	0.09
Emotional Stability	1–5	3.23 (0.86)	3.16 (0.85)	3.23 (0.87)	3.23 (0.84)	3.29 (0.87)	0.003	0.15	0.06
Extraversion	1–5	3.46 (0.88)	3.35 (0.89)	3.47 (0.88)	3.46 (0.88)	3.53 (0.89)	0.005	0.20	0.07
Agreeableness	1–5	3.46 (0.66)	3.47 (0.65)	3.49 (0.67)	3.47 (0.67)	3.42 (0.65)	0.001	−0.08	−0.04
**Cognitive ability**									
Fluid intelligence	0–12	8.84 (2.37)	8.19 (2.56)	8.65 (2.40)	8.98 (2.31)	9.43 (2.07)	0.037	0.54	0.21

*N = 9,300. HISEI: Highest International Socio-Economic Index of occupational status. The table shows the manifest and unweighted sum score for fluid intelligence across all 12 binary matrices and mean scores for the Big Five personality traits. All linear correlations with pSES (column r) are statistically significant at p < 0.01. Likewise, all mean differences (4th quartile vs. 1st quartile) are statistically significant at p < 0.01*.

Examining the mean-level differences of the highest vs. lowest HISEI quartiles provides another way to quantify the personality trait differences we observed. The mean-level differences in the Big Five traits between the highest and lowest quartile of pSES reached standardized effect sizes of up to *d* = 0.20, which is conventionally regarded as a “small” effect.

To facilitate interpretation of the effect sizes, we compared these pSES-related differences in students' levels of the Big Five personality traits with the differences observed in cognitive ability (fluid intelligence). We found a small- to medium-sized correlation between fluid intelligence and parental HISEI, *r* = 0.21, 95% CI [0.19, 0.23], corresponding to the 55th percentile of correlations in individual-differences research (Gignac and Szodorai, 2016). Comparing levels of cognitive ability between the highest vs. lowest HISEI quartiles revealed a mean difference of about half a standard deviation (*d* = 0.54). Hence, pSES-related differences in students' cognitive ability were more than twice as large compared with pSES-related differences in the Big Five.

In addition, we compared the effect sizes ($\eta_{\text{p}}^{2}$) of the pSES-related differences in the Big Five traits with differences related to school track, sex, and migration background. To do so, we conducted analyses of variance in a 4 (HISEI quartiles) × 2 (school track: academically oriented vs. vocationally oriented) × 2 (sex: female vs. male) × 2 (migration background: no vs. yes) between-subjects design. The most sizable differences in the Big Five traits were observed for sex, particularly in relation to Conscientiousness ($\eta_{\text{p}}^{2}$ = 0.023), Openness ($\eta_{\text{p}}^{2}$ = 0.023), and Emotional Stability ($\eta_{\text{p}}^{2}$ = 0.028). Compared with sex-related differences in personality traits, the differences related to pSES, school track, and migration background were small to non-existent (see Supplementary Table 4 for details).

To sum up, personality differed depending on pSES. However, pSES-related differences in personality were substantially smaller than sex-related differences in personality. Moreover, pSES-related differences in personality were smaller than those in cognitive ability.

### Differential Returns to Personality Traits

**Figure 1:**
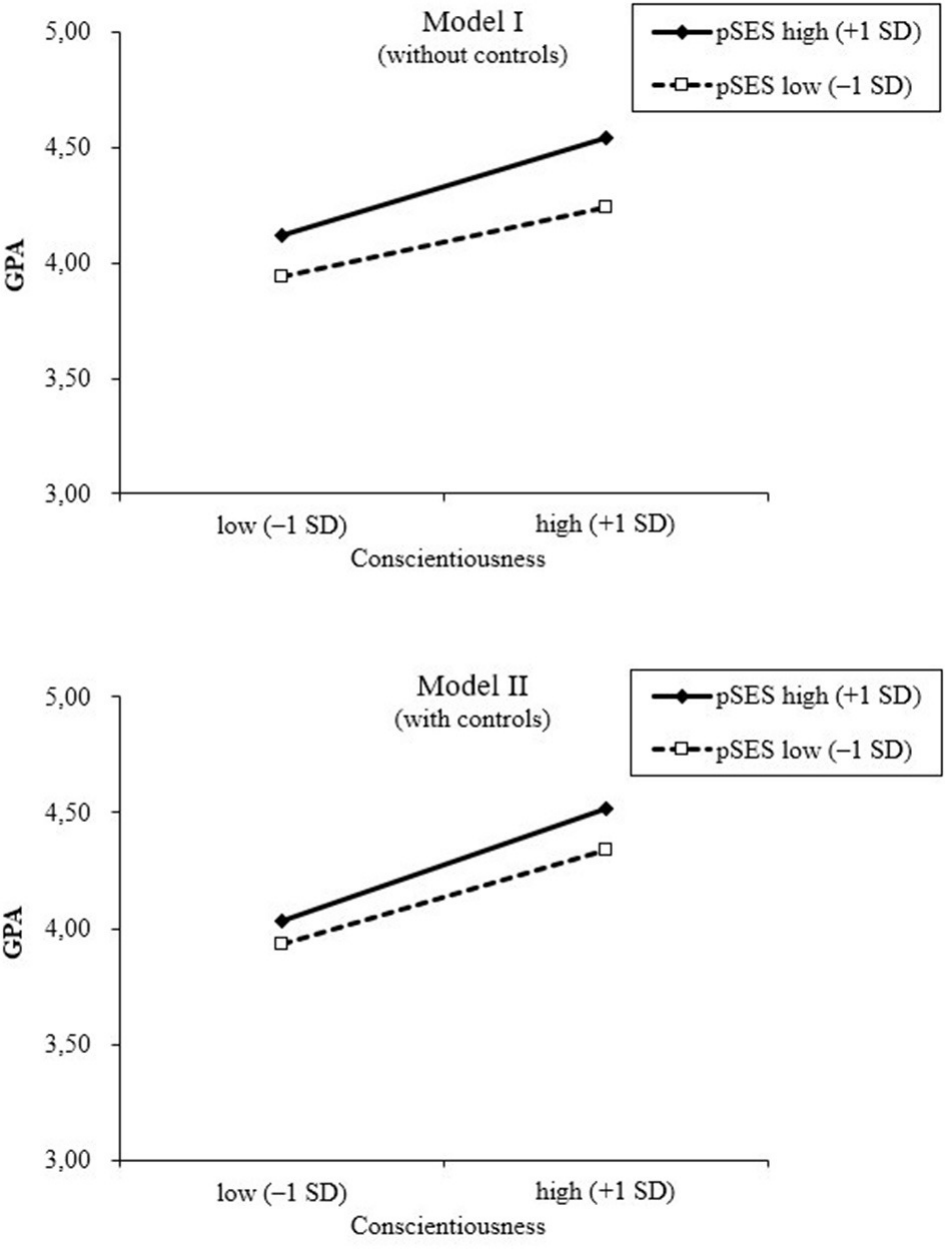
Associations of personality (Conscientiousness) with academic achievement (GPA) by socioeconomic status (HISEI). Model I: Big Five, HISEI, and Big Five × HISEI interactions; Model II: Big Five, HISEI, fluid intelligence, school track, sex, migration background, Big Five × HISEI, Big Five × school track, intelligence × HISEI, and intelligence × school track. Academic achievement was computed as the average across six school subjects (German, math, physics, chemistry, biology, science) of the mid-year report card and was inverted such that higher values corresponded to higher achievement.

Do personality–achievement relations differ depending on pSES? Results from Model I (*R*^2^ = 0.092) revealed that Conscientiousness was positively associated with academic achievement, β = 0.25, 95% CI [0.23, 0.27]; so too, was pSES, β = 0.17, 95% CI [0.15, 0.19]. Importantly, there was a statistically significant interaction between Conscientiousness and parental HISEI, β = 0.05, 95% CI [0.03, 0.07], Δ*R*^2^ = 0.002. Figure 1 illustrates the interaction for students with different levels of pSES. In students whose pSES was high (+1 *SD*), Conscientiousness was more strongly associated with academic achievement, β = 0.30, 95% CI [0.27, 0.33], compared with students with a low pSES (–1 *SD*), β = 0.21, 95% CI [0.18, 0.24].

The interaction between pSES and Conscientiousness in predicting GPA held after including the additional covariates (Model II: *R*^2^ = 0.150; see Table 2 for detailed results). Specifically, the pattern of results remained the same even after controlling for school track and its interactions with personality, β = 0.03, 95% CI [0.01, 0.05], Δ*R*^2^ = 0.001 (see Figure 1).

**Table 2:** Academic achievement regressed on the Big Five personality traits, fluid intelligence, HISEI, school track, sex, and migration background.

	**Model I**			**Model II**		
	**β**	**95% CI**	***p***	**β**	**95% CI**	***p***
Conscientiousness	**0.25**	[0.23, 0.27]	0.000	**0.32**	[0.29, 0.35]	0.000
Openness	**0.03**	[0.01, 0.05]	0.008	0.02	[−0.01, 0.05]	0.124
Emotional Stability	**0.04**	[0.02, 0.06]	0.000	**0.07**	[0.04, 0.10]	0.000
Extraversion	**−0.03**	[−0.05, −0.01]	0.001	**−0.06**	[−0.09, −0.03]	0.000
Agreeableness	**−0.02**	[−0.04, 0.00]	0.035	**−0.06**	[−0.09, −0.03]	0.000
HISEI	**0.17**	[0.15, 0.19]	0.000	**0.10**	[0.08, 0.12]	0.000
Conscientiousness × HISEI	**0.05**	[0.03, 0.07]	0.000	**0.03**	[0.01, 0.05]	0.007
Openness × HISEI	0.00	[−0.02, 0.02]	0.886	0.00	[−0.02, 0.02]	0.769
Emotional Stability × HISEI	−0.01	[−0.03, 0.01]	0.566	−0.02	[−0.04, 0.00]	0.054
Extraversion × HISEI	0.01	[−0.01, 0.03]	0.554	0.02	[0.00, 0.04]	0.092
Agreeableness × HISEI	0.01	[−0.01, 0.03]	0.320	0.02	[−0.01, 0.04]	0.126
Fluid intelligence				**0.26**	[0.22, 0.30]	0.000
School track (0 = academic, 1 = vocational)				**−0.11**	[−0.15, −0.06]	0.000
Sex (0 = female, 1 = male)				0.00	[−0.04, 0.04]	0.997
Migration background (0 = no, 1 = yes)				**−0.14**	[−0.19, −0.09]	0.000
Fluid intelligence × HISEI				0.02	[0.00, 0.04]	0.051
Fluid intelligence × school^a^				**−0.08**	[−0.13, −0.03]	0.002
Conscientiousness × school^a^				**−0.07**	[−0.11, −0.03]	0.002
Openness × school				−0.01	[−0.06, 0.03]	0.507
Emotional Stability × school^a^				**−0.07**	[−0.11, −0.02]	0.002
Extraversion × school^a^				**0.07**	[0.03, 0.11]	0.001
Agreeableness × school^a^				**0.06**	[0.01, 0.10]	0.010
*R^2^*	**0.092**		0.000	**0.150**		0.000

*N = 9,300. Model I: Big Five, HISEI and Big Five × HISEI interactions; Model II: Big Five, HISEI, fluid intelligence, school track, sex, migration background, Big Five × HISEI, Big Five × school track, intelligence × HISEI, and intelligence × school track. HISEI: Highest International Socio-Economic Index of occupational status. Significant model parameters (p < 0.05) are in bold print*.

a *See Supplementary Figures 1–5 for interaction plots*.

The associations of the other Big Five dimensions with academic achievement were small (β = −0.03–0.04). Moreover, none of the other Big Five dimensions had differential associations with academic achievement depending on pSES, as evident from the near-zero interaction effects. Thus, only for Conscientiousness but none of the other traits did trait–achievement relations differ.

Finally, we compared differential returns to personality with differential returns to cognitive ability. Our results revealed, first, that the strength of the association of Conscientiousness with academic achievement (β = 0.32) was comparable with that of fluid intelligence (β = 0.26). Second, and in contrast to Conscientiousness, the relation between fluid intelligence and academic achievement did not depend on pSES, β = 0.02, 95% CI [0.00, 0.04].

## Discussion

Socio-emotional skills such as the Big Five personality traits have received increased attention as a potential target for interventions aimed at improving students' academic outcomes and reducing the achievement gaps between students from diverse socio-economic backgrounds (e.g., Kautz et al., 2014; Sánchez Puerta et al., 2016; Chernyshenko et al., 2018; Bleidorn et al., 2019; Malanchini et al., 2019). However, whether personality traits are an apt target for intervention remains unclear, because the interplay between pSES, personality traits, and academic achievement is poorly understood. In this study, we added to this debate by examining two forms of social inequality in personality: (1) differences in students' *levels* of personality traits depending on pSES; and (2) differences in the *returns* to personality traits in terms of differential personality–achievement relationships depending on pSES. Our results partly replicate and partly deviate from previous findings.

Differences in the levels of personality traits related to students' pSES were small—and clearly smaller than gender differences in personality and differences in cognitive ability depending on pSES, which we took as a reference point. The largest pSES-related differences emerged for Openness to Experience and Conscientiousness, which happen to be the two Big Five traits that are most strongly related to academic achievement (Poropat, 2009), including in Germany (Lechner et al., 2017). Specifically, higher-SES students reported higher levels of Openness (*r* = 0.09). A correlation of this size corresponds roughly to the 20th percentile of all effect sizes observed in individual differences research according to the meta-analytically derived guidelines proposed by Gignac and Szodorai (2016). This correlation replicates the pSES-related differences in Openness reported in the recent meta-analysis by Ayoub et al. (2018) and a large-scale study by Sutin et al. (2017). The extent to which the pSES–Openness association reflects social causation (e.g., Sutin et al., 2017) or heritability (e.g., Mõttus et al., 2019) deserves further scrutiny in future work.

With regard to Conscientiousness, higher-SES students reported lower (not higher) levels of Conscientiousness than their lower-SES peers (*r* = −0.07). This finding differs from Ayoub et al. (2018) but replicates a finding by Sutin et al. (2017) in the younger samples (age ca. 14–30 years) and more recent cohorts that they analyzed. The slightly lower levels of Conscientiousness among high-SES students are at odds with the idea that a higher SES favors the development of Conscientiousness (family investment model; Conger and Donnellan, 2007) or that low SES impairs the development of Conscientiousness because lower-SES families cannot provide children with learning opportunities or stimulation comparable with those provided by higher-SES families (family stress model; Conger and Conger, 2002). One can speculate that parents working in jobs with lower occupational prestige might foster diligence and a will to achieve in their offspring, possibly because their lower-status positions require such traits more than higher-status positions do.

Notably, the lack of major pSES-related differences in the levels of personality traits are unlikely to have been an artifact of the measures used in NEPS. By comparing pSES-related differences in the Big Five traits with the—often sizable—sex-related differences in the same traits, we established that the Big Five traits do not, in general, lack sensitivity for potential differences between sociodemographic subgroups. In turn, the considerable differences in cognitive ability between students with different pSES as measured by HISEI show that social inequality in cognitive ability was much stronger than that in personality traits.

With regard to social inequality in the returns to personality, we found only limited evidence that personality–achievement relations differ depending on students' pSES. The only statistically significant interaction was pSES × Conscientiousness: Conscientiousness was more strongly related to GPA among students from higher-SES families (although higher-SES students, on average, reported slightly lower levels of Conscientiousness than students from lower-SES families). This finding is in line with the resource amplification hypothesis, but it deviates from the results obtained by Ayoub et al. (2018, Study 2), who found support for the resource substitution hypothesis (i.e., the grades of lower-SES students in a large but likely non-representative online sample benefitted more from higher Conscientiousness). Our results suggest that students from higher-SES backgrounds gain more than lower-SES students from the same levels of Conscientiousness in terms of better academic achievement. This *pSES* × *Conscientiousness* interaction shrank but still held even after controlling for students' school tracks and the interaction between personality and school track. As we explained earlier, school tracks are a key conduit for the intergenerational transmission of SES and achievement/attainment in the historically three-tiered German school system (e.g., Baumert et al., 2006; Maaz et al., 2008). Our results suggest that the *pSES* × *Conscientiousness* interaction is partly independent of school track, which Brandt et al. (2020) found to be a moderator of trait–achievement relations in the German school system. As was the case with the *pSES* × *Conscientiousness* interaction in the present study, school track in the Brandt et al. (2020) study moderated trait–achievement relations largely as the resource amplification hypothesis would predict (personality traits were more strongly related to achievement in the higher school tracks).

The mechanisms behind the *pSES* × *Conscientiousness* interaction in our study and other *pSES* × *personality* interactions in earlier studies (e.g., Damian et al., 2015; Ayoub et al., 2018) as well as the *school track* × *personality* interactions observed by Brandt et al. (2020) have yet to be uncovered. Such interactions may indicate resource substitution or amplification; but they may also reflect differential trait activation (Tett and Burnett, 2003) depending on school context (e.g., social composition of students; achievement-related norms and expectations). School contexts differ for higher- and lower-SES students because of pSES-related selection into different school types. For example, students with a lower pSES are often selected into lower, vocationally oriented tracks (Maaz et al., 2008) that demand and promote Conscientiousness less than higher, academically oriented tracks do (Brandt et al., 2020). It may also be the case that teachers' grading practices, and especially the extent to which they reward certain student traits, differ depending on students' pSES and/or track. Future research should explore these possibilities. Overall, however, the almost complete absence of *pSES* × *personality* interactions supports the “independent effects hypothesis” (Damian et al., 2015), which states that pSES and personality traits largely operate as independent resources.

## Limitations

Three limitations of our study should be mentioned. First, its correlational design precludes causal interpretations. For example, the pSES–trait and pSES–achievement relations cannot be unequivocally interpreted as reflecting *social* causation. There might be unobserved confounders such as shared genetic influences behind these relationships (e.g., Tucker-Drob et al., 2016; Mõttus et al., 2019). Moreover, our study's cross-sectional design meant that we could not untangle the temporal dynamics of the interplay between pSES, socio-emotional skills, and achievement. Future studies could gain additional insights by tracing this interplay from early childhood into adolescence.

Second, we only had an ultra-short measure of personality at our disposal. Research shows that short scales work better than often assumed. For example, the 10-item BFI-10 and the 15-item BFI-2-XS largely reproduce trait–outcome relations of (often much) longer scales (e.g., Thalmayer et al., 2011; Rammstedt et al., 2020). Reassuringly, the observed pSES–trait relations were largely in line with those found in the recent meta-analysis by Ayoub et al. (2018) and the study by Sutin et al. (2017). Moreover, it is beneficial that the BFI-10 controls for acquiescence, which is a likely source of bias in the pSES–personality association because acquiescence is higher in individuals with lower education (Rammstedt et al., 2010) and lower cognitive ability (Lechner and Rammstedt, 2015). However, ultra-short measures such as the BFI-10 do not allow for facet-level analyses, which are often a more promising level of abstraction in trait–outcome research (e.g., Danner et al., 2020; Mõttus et al., 2020). Hence, it would be desirable for future research to revisit the interplay between pSES, socio-emotional skills, and achievement using faceted measures of personality. Future research should also move beyond personality self-reports and include observer ratings to probe pSES-related differences in parent- and/or teacher-reported personality. This may allow for additional insights, for example, into whether teachers rate students' personalities differently than students themselves depending on students' pSES (i.e., whether there are discrepancies between teachers' perceptions and students self-concepts depending on pSES, e.g., because of stereotyping).

Third, our analyses focused on secondary school students in Germany. Although the links between pSES and the Big Five traits that we found were comparable with those reported in prior research from other (mostly Western) countries, the interplay between pSES, school tracks, personality, and achievement might depend more strongly on the institutional (e.g., the tracking system) and cultural (e.g., norms and expectations regarding what constitutes desirable personality traits) makeup of a country. Cross-nationally comparative research could therefore arrive at a better understanding of the role of context in the interplay between pSES, personality, and achievement.

## Conclusion

Our findings suggest that social inequality in both the levels of personality traits and the returns to personality traits is limited. There were no or only small pSES-related personality differences among students. The largest correlation of pSES was that with Openness (*r* = 0.09). We also found that the personality–achievement relations did not depend on pSES—with one important exception: Students from lower-SES families did not benefit from their Conscientiousness as much as students from higher-SES families did. This effect appeared to be small, but it was at least partly independent of the school tracks that students attended. The latter result adds a cautionary note for researchers and practitioners who hope that fostering socio-emotional skills especially among socially disadvantaged students might be a viable strategy to reduce inequality in achievement. Lower-SES students were neither at a disadvantage when it came to Conscientiousness, nor did they benefit from higher levels of Conscientiousness as much as their higher-SES peers did. This does not rule out the possibility that fostering traits such as Openness or Conscientiousness benefits students' achievement, but it does cast some doubt on whether this would reduce inequality in achievement.

## Data Availability Statement

Publicly available datasets were analyzed in this study. This paper used data from the National Educational Panel Study (NEPS): Starting Cohort 4–9th Grade, doi: 10.5157/NEPS:SC4:10.0.0. From 2008 to 2013, NEPS data were collected as part of the Framework Programme for the Promotion of Empirical Educational Research funded by the German Federal Ministry of Education and Research (BMBF). As of 2014, the NEPS survey is carried out by the Leibniz Institute for Educational Trajectories (LIfBi) at the University of Bamberg in cooperation with a nationwide network.

## Ethics Statement

Ethical approval was not provided for this study on human participants because this paper uses secondary data from the German National Educational Panel Study (NEPS). All data collections that took place as part of NEPS were reviewed and approved under German law and research ethics codes. Written informed consent to participate in this study was provided by the participants' legal guardian/next of kin.

## Author Contributions

CL: funding acquisition (lead), conceptualization, methodology, project administration, writing—original draft, and writing—review & editing. JB: funding acquisition, conceptualization, data curation, formal analysis, methodology, visualization, and writing—original draft. NB: conceptualization, methodology, and writing—review & editing. BR: funding acquisition, conceptualization, and writing—review & editing. All authors contributed to the article and approved the submitted version.

## Conflict of Interest

The authors declare that the research was conducted in the absence of any commercial or financial relationships that could be construed as a potential conflict of interest.
