# Supplementary material for: Two Forms of Social Inequality in Students' Socio-Emotional Skills: Do the Levels of Big Five Personality Traits and Their Associations With Academic Achievement Depend on Parental Socioeconomic Status?
Source: Front Psychol. 2021 Jul 21;12:679438. doi: 10.3389/fpsyg.2021.679438 (PMC8335486; doi:10.3389/fpsyg.2021.679438)
Supplement: Supplementary file 1 [file Table_1.docx]

Supplementary Online Material

for the article

Lechner, C. M., Bender, J., Brandt, N. D., & Rammstedt, B. (2021). Two Forms of Social Inequality in Students’ Socio-Emotional Skills: Do the Levels of Big Five Personality Traits and Their Associations with Academic Achievement Depend on Parental Socioeconomic Status? *Frontiers in Psychology.* doi: 10.3389/fpsyg.2021.679438

# Supplementary Table 1

*Descriptive Statistics and Correlations for Study Variables*

| Variable | Range | *M* | *SD* | Internal consistency | 1 | 2 | 3 | 4 | 5 | 6 | 7 | 8 |
| --- | --- | --- | --- | --- | --- | --- | --- | --- | --- | --- | --- | --- |
| 1. Conscientiousness | 1 – 5 | 3.17 | 0.88 | 0.56 | — |  |  |  |  |  |  |  |
| 2. Openness | 1 – 5 | 3.50 | 0.94 | 0.44 | **0.08** | — |  |  |  |  |  |  |
| 3. Emotional stability | 1 – 5 | 3.23 | 0.86 | 0.43 | **−0.04** | −0.01 | — |  |  |  |  |  |
| 4. Extraversion | 1 – 5 | 3.46 | 0.88 | 0.66 | 0.01 | **0.07** | **0.25** | — |  |  |  |  |
| 5. Agreeableness | 1 – 5 | 3.46 | 0.66 | 0.40 | **0.22** | **0.13** | −0.02 | **−0.07** | — |  |  |  |
| 6. HISEI | 10 – 90 | 51.63 | 20.58 | — | **−0.07** | **0.09** | **0.06** | **0.07** | **−0.04** | — |  |  |
| 7. GPA | 1 – 6 | 4.21 | 0.71 | 0.87 | **0.24** | **0.06** | **0.03** | −0.01 | **0.03** | **0.15** | — |  |
| 8. Fluid intelligence | 0 – 12 | 8.84 | 2.37 | 0.66 | **−0.10** | **0.07** | **0.03** | **−0.05** | 0.01 | **0.21** | **0.23** | — |

*Note*. *N* = 9,300. HISEI: Highest International Socio-Economic Index of occupational status. Descriptive statistics and correlations are based on manifest variables and unweighted data. GPA was computed as the average across six school subjects (German, math, physics, chemistry, biology, science) of the mid-year report card and were inverted such that higher values corresponded to higher achievement. To provide estimates of internal consistency, we report the Spearman-Brown coefficient for the Big Five (as is appropriate for 2-item measures) and Cronbach’s alpha for GPA and fluid intelligence. Significant correlations (*p* < .05) are in bold print.

# Structural Validity of the Big Five Personality Measure

We estimated a series of multiple‐group measurement models to test the structural validity of the BFI-10. More precisely, we modeled the Big Five personality traits as five obliquely rotated exploratory structural equation modeling factors (ESEM) across the 11 Big Five items (plus an acquiescence factor). For more details on exploratory structural equation modeling, see Marsh, Morin, Parker, and Kaur (2014). Moreover, we modeled socio-economic status (HISEI quartiles) as a grouping variable to test the measurement invariance and thus comparability of the BFI-10 across socio-economic status groups. We estimated measurement models for configural, metric, scalar, strict, and partial scalar measurement invariance and compared the models in terms of model fit. We judged model fit by the comparative fit index (CFI), Tucker–Lewis index (TLI), root mean square error of approximation (RMSEA), and standardized root mean square residual (SRMR). We estimated all models using a robust maximum likelihood estimator (MLR) and applied longitudinal sampling weights, thus accounting for unequal sampling probabilities and longitudinal attrition between Waves 1 and 2. Our analyses revealed that even partial scalar invariance held for the BFI-10, i.e. equal factor loadings for all items and equal intercepts for most items. We then used the parameter estimates from the final ESEM solution (partial scalar) as starting values to estimate the ESEM-within-CFA model. Additionally, we assumed equal variances of the Big Five dimensions across the HISEI quartiles. The ESEM-within-CFA model showed good model fit. For detailed results of these analyses, including factor loadings and model fit for different levels of measurement invariance, see Supplementary Tables 2 and 3.

# Supplementary Table 2

*Loadings of the BFI-10+1 Items on the Big Five Dimensions*

| **Item** | **Ope** | **Con** | **Ext** | **Agr** | **Emo** |
| --- | --- | --- | --- | --- | --- |
| I have few artistic interests (Ope–) | **–.48** | -.00 | .07 | –.03 | .00 |
| I have an active imagination (Ope+) | **.71** | –.14 | .00 | –.00 | .12 |
| I am an idler, tend to be lazy (Con–) | .00 | **–.71** | –.00 | .01 | .15 |
| I do a thorough job (Con+) | .07 | **.55** | –.09 | .01 | .00 |
| I am rather restrained, reserved (Ext–) | .01 | .00 | **–.68** | .17 | .00 |
| I am outgoing, sociable (Ext+) | .01 | .04 | **.70** | .00 | .01 |
| I tend to find fault with others (Agr–) | .00 | –.22 | .12 | **–.46** | –.06 |
| I am considerate of others, sensitive (Agr+) | .20 | .19 | .00 | **.40** | –.00 |
| I am generally trusting, I believe people are good (Agr+) | .03 | –.00 | .13 | **.35** | –.04 |
| I get nervous easily (Emo–) | –.00 | –.12 | –.23 | .17 | **–.49** |
| I am relaxed, handle stress well (Emo+) | –.01 | –.02 | .00 | .18 | **.61** |

*Note.* Y-standardized loadings from the ESEM-within-CFA model for the first HISEI quartile (values for the other three quartiles differed only minimally due to differences in the manifest item variances). Highest loadings defining each factor are in bold print. Ope: Openness; Con: Conscientiousness; Ext: Extraversion; Agr: Agreeableness; Emo: Emotional Stability.

# Supplementary Table 3

*Model Fit Indices from the Measurement Models of Socio-Emotional Skills (Big Five) Testing Measurement Invariance Across Socioeconomic Status Groups (HISEI Quartiles)*

| Model | χ^2^ (*df*) | RMSEA | CFI | TLI | SRMR |
| --- | --- | --- | --- | --- | --- |
| Configural | 108.038(36) | .027 | .992 | .948 | 0.008 |
| Metric | 177.837(126) | .012 | .994 | .989 | 0.015 |
| Scalar | 386.389(159) | .023 | .973 | .963 | 0.026 |
| Strict | 466.128(192) | .023 | .968 | .963 | 0.038 |
| Partial scalar 1  (Con–) | 355.400(156) | .022 | .977 | .967 | 0.025 |
| Partial scalar 2  (Con–, Con+) | 329.328(153) | .021 | .979 | .970 | 0.024 |
| Partial scalar 3  (Con–, Con+, Agr–) | 295.355(150) | .019 | .983 | .975 | 0.022 |
| Partial scalar 4  (Con–, Con+, Agr–, Ope–) | 267.655(147) | .018 | .989 | .979 | 0.021 |
| ESEM-within-CFA  (equal variances)  (Con–, Con+, Agr–, Ope–) | 207.401(144) | .013 | .993 | .989 | 0.019 |

*Note*. For the partial scalar invariance models, the equality constraints on the intercepts of various BFI-10+1 items were relaxed, whereas all other constraints were retained.

# Supplementary Table 4

*Effect Sizes (η_p_^2^) of the Mean-Level Differences in Personality (Big Five) and Cognitive Ability (Fluid Intelligence) by Socioeconomic Status (HISEI), School Track, Sex, and Migration Background*

|  | Con | Ope | Emo | Ext | Agr | Int |
| --- | --- | --- | --- | --- | --- | --- |
|  | η_p_^2^ | η_p_^2^ | η_p_^2^ | η_p_^2^ | η_p_^2^ | η_p_^2^ |
| HISEI (quartiles) | .000 | **.003** | **.002** | **.003** | **.001** | **.002** |
| School track  (0 = academic, 1 = non-academic) | **.008** | .000 | .000 | .000 | .000 | **.078** |
| Sex (0 = female, 1 = male) | **.023** | **.023** | **.028** | **.001** | **.006** | **.003** |
| Migration background  (0 = no, 1 = yes) | .000 | **.002** | .000 | .000 | .000 | **.004** |
| HISEI × school track | .001 | .000 | **.001** | .000 | .001 | .000 |
| HISEI × sex | .000 | .001 | .000 | .000 | .000 | .001 |
| HISEI × migration background | .000 | .000 | .000 | .000 | **.001** | .000 |
| School track × sex | **.000** | .000 | .000 | .000 | .000 | .000 |
| School track × migration background | .000 | .000 | .000 | .000 | **.001** | **.001** |
| Sex × migration background | .000 | .000 | .000 | .000 | .000 | **.001** |
| HISEI × school track × sex | .000 | .001 | .000 | .000 | .001 | .000 |
| HISEI × school track × migration background | .000 | .000 | .000 | .000 | .001 | .000 |
| HISEI × sex × migration background | .000 | **.001** | **.001** | .000 | .001 | .000 |
| School track × sex × migration background | .000 | .000 | .000 | .000 | .000 | .000 |
| HISEI × school track × sex × migration background | .000 | .000 | .000 | .000 | .000 | .000 |

*Note. N* = 9,300. Con: Conscientiousness; Emo: Emotional Stability; Ope: Openness; Ext: Extraversion; Agr: Agreeableness; Int: Fluid intelligence; HISEI: Highest international socio-economic index of occupational status. The table shows effect sizes (η_p_^2^) from separate ANOVAs (one for each Big Five dimension and one for fluid intelligence). Significant effects (*p* < .05) are in bold print.

# Differential Associations by School Track

Our main research question regarding differential returns to socio-emotional skills focused on social inequality by socioeconomic status. Therefore, we analyzed differential associations of the Big Five personality traits – particularly Conscientiousness – with academic achievement (GPA) depending on students’ socioeconomic status. However, our analyses also revealed differential associations by school track, more precisely for Conscientiousness, Emotional Stability, Extraversion, and Agreeableness (also see Table 2 of the Main Document). That is, Conscientiousness was more strongly associated with achievement in academic school tracks, β = 0.32, 95% CI [0.29, 0.35], compared to non-academic school tracks, β = 0.25, 95% CI [0.22, 0.28], see Supplementary Figure 1. Moreover, Emotional Stability was positively associated with achievement in academic school tracks, β = 0.07, 95% CI [0.04, 0.10], but unrelated to achievement in non-academic school tracks, β = 0.01, 95% CI [–0.02, 0.03], see Supplementary Figure 2. Extraversion was negatively associated with achievement in academic school tracks, β = –0.06, 95% CI [–0.09, –0.02], but unrelated to achievement in non-academic school tracks, β = 0.01, 95% CI [–0.01, 0.04], see Supplementary Figure 3. Likewise, Agreeableness was negatively associated with achievement in academic school tracks, β = –0.06, 95% CI [–0.09, –0.03], but unrelated to achievement in non-academic school tracks, β = 0.00, 95% CI [–0.03, 0.02], see Supplementary Figure 4. Beyond personality, we found differential associations for fluid intelligence by school track. That is, fluid intelligence was more strongly associated with achievement in academic school tracks, β = 0.26, 95% CI [0.22, 0.30], compared to non-academic school tracks, β = 0.18, 95% CI [0.16, 0.21], see Supplementary Figure 5. These findings are largely in line with previous research showing differential associations of personality and cognitive ability with academic achievement across school tracks (Brandt et al., 2020).

# Supplementary Figure 1

*Associations of Personality (Conscientiousness) with Academic Achievement (School Grades) by School Track*


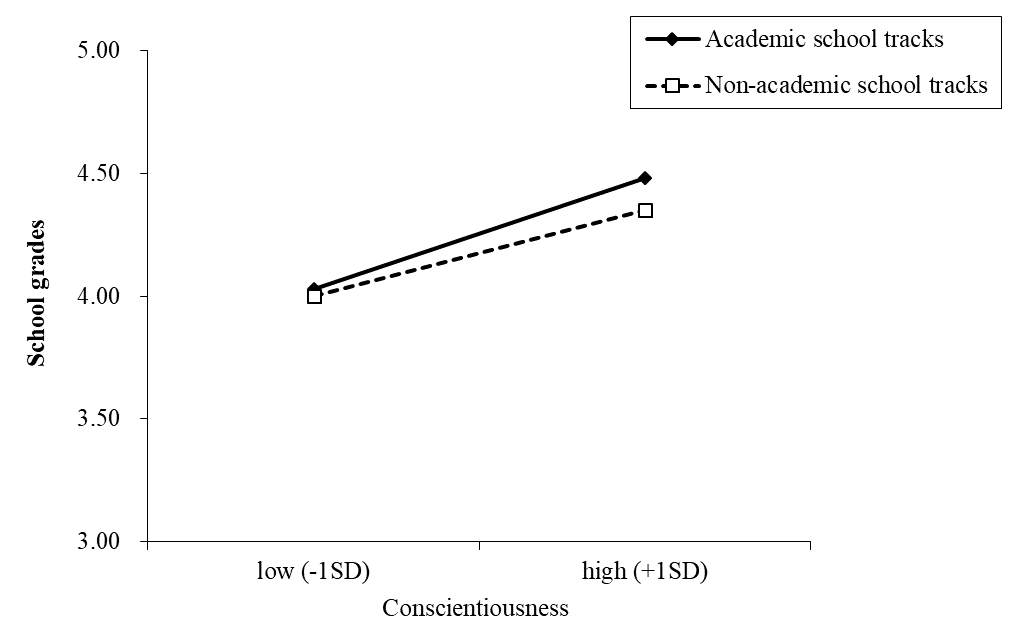


*Note.* Predicted values are based on Model II (Big Five, HISEI, fluid intelligence, school track, sex, migration background, Big Five × HISEI, Big Five × school track, intelligence × HISEI, and intelligence × school track). School grades were computed as the average across six school subjects (German, Math, Physics, Chemistry, Biology, Natural Sciences) of the mid-year report card and were inverted such that higher values correspond to higher achievement.

# Supplementary Figure 2

*Associations of Personality (Emotional Stability) with Academic Achievement (School Grades) by School Track*


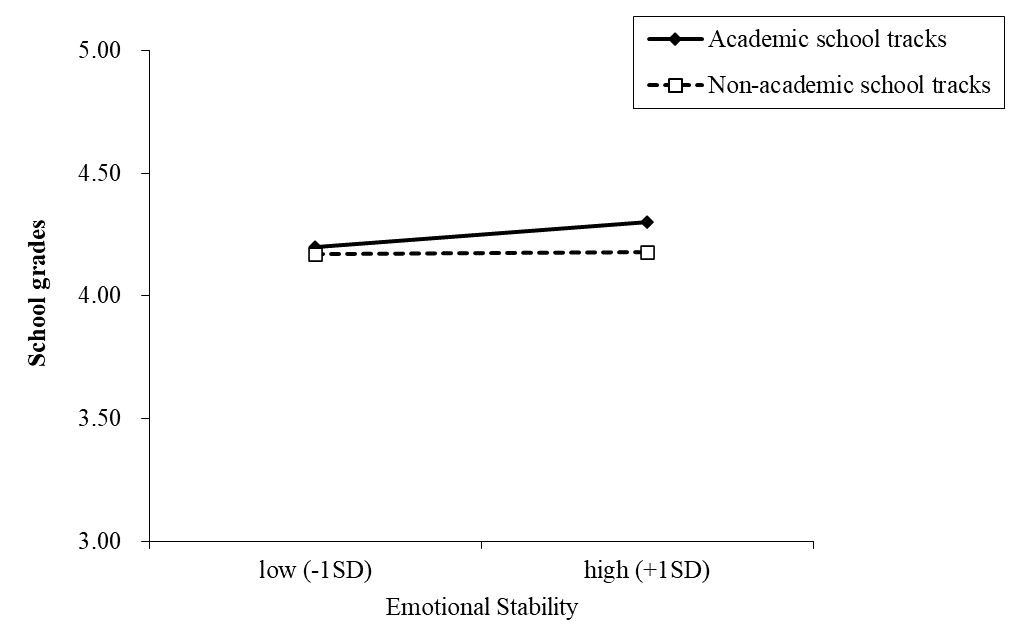


*Note*. Predicted values are based on Model II (Big Five, HISEI, fluid intelligence, school track, sex, migration background, Big Five × HISEI, Big Five × school track, intelligence × HISEI, and intelligence × school track). School grades were computed as the average across six school subjects (German, Math, Physics, Chemistry, Biology, Natural Sciences) of the mid-year report card and were inverted such that higher values correspond to higher achievement.

# Supplementary Figure 3

*Associations of Personality (Extraversion) with Academic Achievement (School Grades) by School Track*


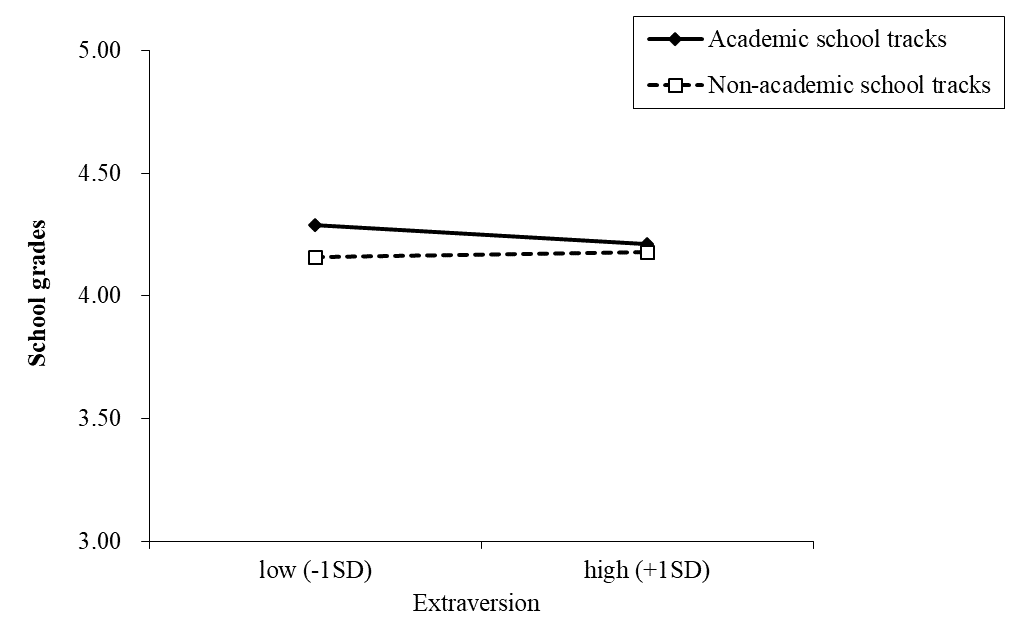


*Note*. Predicted values are based on Model II (Big Five, HISEI, fluid intelligence, school track, sex, migration background, Big Five × HISEI, Big Five × school track, intelligence × HISEI, and intelligence × school track). School grades were computed as the average across six school subjects (German, Math, Physics, Chemistry, Biology, Natural Sciences) of the mid-year report card and were inverted such that higher values correspond to higher achievement.

# Supplementary Figure 4

*Associations of Personality (Agreeableness) with Academic Achievement (School Grades) by School Track*


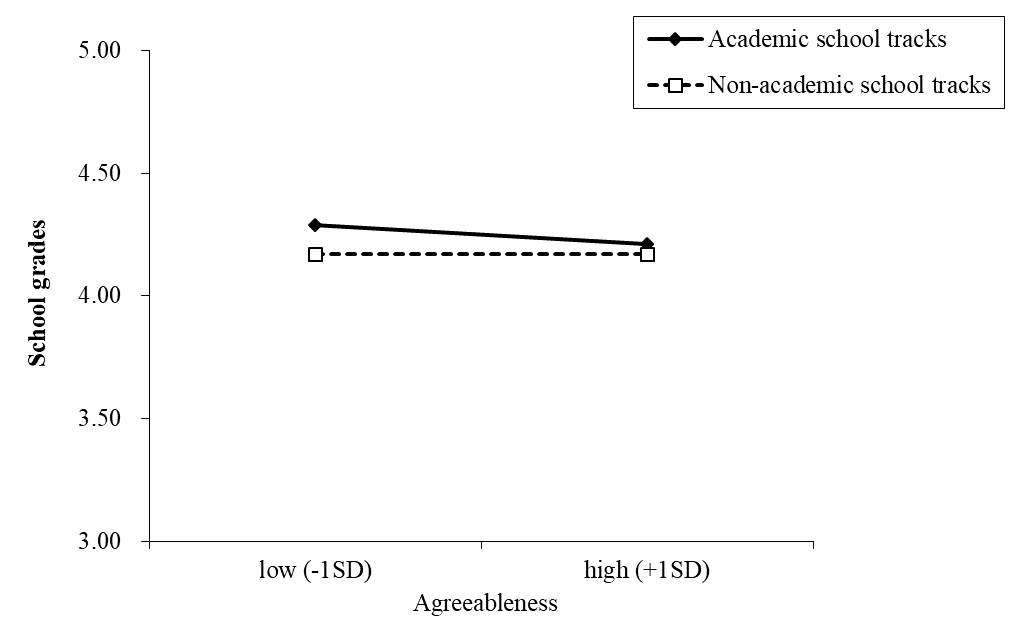


*Note*. Predicted values are based on Model II (Big Five, HISEI, fluid intelligence, school track, sex, migration background, Big Five × HISEI, Big Five × school track, intelligence × HISEI, and intelligence × school track). School grades were computed as the average across six school subjects (German, Math, Physics, Chemistry, Biology, Natural Sciences) of the mid-year report card and were inverted such that higher values correspond to higher achievement.

# Supplementary Figure 5

*Associations of Cognitive Ability (Fluid Intelligence) with Academic Achievement (School Grades) by School Track*


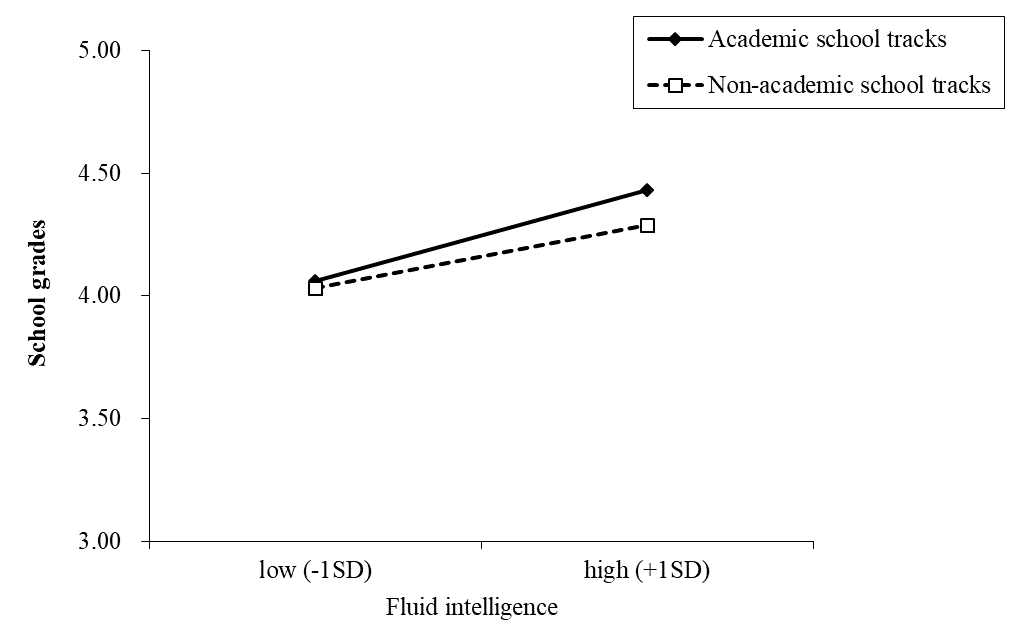


*Note.* Predicted values are based on Model II (Big Five, HISEI, fluid intelligence, school track, sex, migration background, Big Five × HISEI, Big Five × school track, intelligence × HISEI, and intelligence × school track). School grades were computed as the average across six school subjects (German, Math, Physics, Chemistry, Biology, Natural Sciences) of the mid-year report card and were inverted such that higher values correspond to higher achievement.

# References

Brandt, N. D., Lechner, C. M., Tetzner, J., & Rammstedt, B. (2020). Personality, cognitive ability, and academic performance: Differential associations across school subjects and school tracks. *Journal of Personality*, *88*(2), 249–265. https://doi.org/10.1111/jopy.12482

Marsh, H. W., Morin, A. J. S., Parker, P. D., & Kaur, G. (2014). Exploratory Structural Equation Modeling: An Integration of the Best Features of Exploratory and Confirmatory Factor Analysis. *Annual Review of Clinical Psychology, 10*(1), 85–110. https://doi.org/10.1146/annurev-clinpsy-032813-153700
